# An Exaggerated Immune Response in Female BALB/c Mice Controls Initial *Toxoplasma gondii* Multiplication but Increases Mortality and Morbidity Relative to Male Mice

**DOI:** 10.3390/pathogens10091154

**Published:** 2021-09-08

**Authors:** Rasha Alonaizan, Stuart Woods, Kerrie E Hargrave, Craig W. Roberts

**Affiliations:** 1 Strathclyde Institute of Pharmacy & Biomedical Sciences, University of Strathclyde, 161 Cathedral Street, Glasgow G4 0RE, UK; ralonezan@ksu.edu.sa (R.A.); stuart.woods@strath.ac.uk (S.W.); Kerrie.Hargrave@glasgow.ac.uk (K.E.H.); 2 Faculty of Science, King Saud University, P.O. Box 2455, Riyadh 11451, Saudi Arabia; 3 Centre for Immunobiology, Institute of Infection, Immunity and Inflammation, 120 University Place, University of Glasgow, Glasgow G12 8QQ, UK

**Keywords:** *Toxoplasma gondii*, sex differences, immune response, IVIS, parasite burdens, MCP-1, immune endocrine

## Abstract

Studies indicate that female mice are more susceptible to *T. gondii* infection, as defined by higher mortality rates in comparison to male mice. However, whether this is due to an inability to control initial parasite multiplication or due to detrimental effects of the immune system has not been determined. Therefore, the following studies were undertaken to determine the influence of sex on early parasite multiplication and the immune response during *T. gondii* infection and to correlate this with disease outcome. Early parasite replication was studied through applying an in vivo imaging system (IVIS) with luciferase expressing *T. gondii*. In parallel immunological events were studied by cytometric bead array to quantify key immunological mediators. The results confirmed the previous findings that female mice are more susceptible to acute infection, as determined by higher mortality rates and weight loss compared with males. However, conflicting with expectations, female mice had lower parasite burdens during the acute infection than male mice. Female mice also exhibited significantly increased production of Monocyte Chemoattractant Protein-1 (MCP-1), Interferon (IFN)-γ, and Tumour Necrosis Factor (TNF)-α than male mice. MCP-1 was found to be induced by *T. gondii* in a dose dependent manner suggesting that the observed increased levels detected in female mice was due to a host-mediated sex difference rather than due to parasite load. However, MCP-1 was not affected by physiological concentration of estrogen or testosterone, indicating that MCP-1 differences observed between the sexes in vivo are due to an as yet unidentified intermediary factor that in turn influences MCP-1 levels. These results suggest that a stronger immune response in female mice compared with male mice enhances their ability to control parasite replication but increases their morbidity and mortality.

## 1. Introduction

*Toxoplasma gondii (T. gondii)* is the most successful intracellular parasitic organism and its distribution is worldwide. It is capable of infecting all warm-blooded animals, including humans [1]. *T. gondii* infects about one-third of the world’s population in all continents. Based on clinical observations the vast majority of immune-competent individuals infected with *T. gondii* are asymptomatic or experience mild self-limiting illness [2]. However, highly virulent strains of *T. gondii* can cause severe ocular disease even in immune-competent adults [1]. While immunocompromised individuals, such as AIDS patients, may experience toxoplasmic encephalitis associated with infection, *T. gondii* is also medically important in other immunocompromised individuals such as patients undertaking cancer therapy or organ transplantation [2]. If a woman is infected during pregnancy, *T. gondii* may cause congenital toxoplasmosis, abortion, and neonatal mortality [3].

Immunity against *T. gondii* needs early production of the pro-inflammatory cytokines like IL-12 that stimulates natural killer (NK) cells, CD4+ T cells, and CD8+ T cells to produce IFN-γ [4]. IL-12 and IFN-γ are the key cytokines during the immune response to *T. gondii* due to their role in promoting parasite death, controlling tachyzoite population load and tachyzoite-bradyzoite developmental conversion [5]. Furthermore, IL-12 plays a key role in directing the adaptive immune response, including the differentiation of Th1 cells [6]. IFN-γ production stimulates a range of antimicrobial events within the host cell including modification of host cell tryptophan metabolism, iron starvation, and induction of nitric oxide (NO) that regulates intracellular parasite proliferation [4,7].

The vast majority of humans with *T. gondii* are asymptomatic. However, a number of clinical studies in humans, have reported sex differences in disease manifestations. Lymphadenopathy was more frequently observed in males than in females under the age of 15 years. In contrast, in people over the age of 25 years, lymphadenopathy was more commonly reported in females [8]. Furthermore, toxoplasmic encephalitis has been reported to be more likely an AIDS-defining disease in females compared to males [9]. Early studies, demonstrated that female mice develop worse inflammation in their brain than male mice following infection with *T. gondii*. While gonadectomy reduced disease severity, estrogen administration increased disease severity [10,11].

Studying a wide variety of inbred mice of different genetic backgrounds and major histocompatibility complex haplotypes revealed that female mice generally had increased mortality compared with male mice following *T. gondii* infection [12]. Subsequent studies also found female severe combined immunodeficient (SCID) mice to have increased pathology in their brains and quantitatively different immune responses compared with male mice [13]. These studies indicate that early innate immune events are different between the sexes and are likely determinants of disease outcome. Importantly, these early studies did not measure parasite multiplication during the acute stages of infection or look at levels of many circulating immune mediators. These omissions, largely due to technological limitations at the time, are important since mortality in mice can be due to an inability to control parasite replication or through uncontrolled inflammation as exemplified in IL-4 and IL-10 deficient mice that succumb due to cytokine shock [14,15]. However, since the original studies described above, advances in technology should now allow for a reappraisal of this situation.

The studies described herein make use of more recently available technology to further our knowledge of sex differences of mice in the response to *T. gondii* infection. Specifically, we use an in vivo imaging system (IVIS) and luciferase expressing *T. gondii* parasites to determine the role of sex in early parasite multiplication and a cytometric bead array to determine potential differences in multiple diverse immunological mediators in male and female BALB/c mice infected with *T. gondii*.

## 2. Results

### 2.1. Female Mice Increased Mortality Following T. gondii Infection Compared to Male Mice

**Figure 1:**
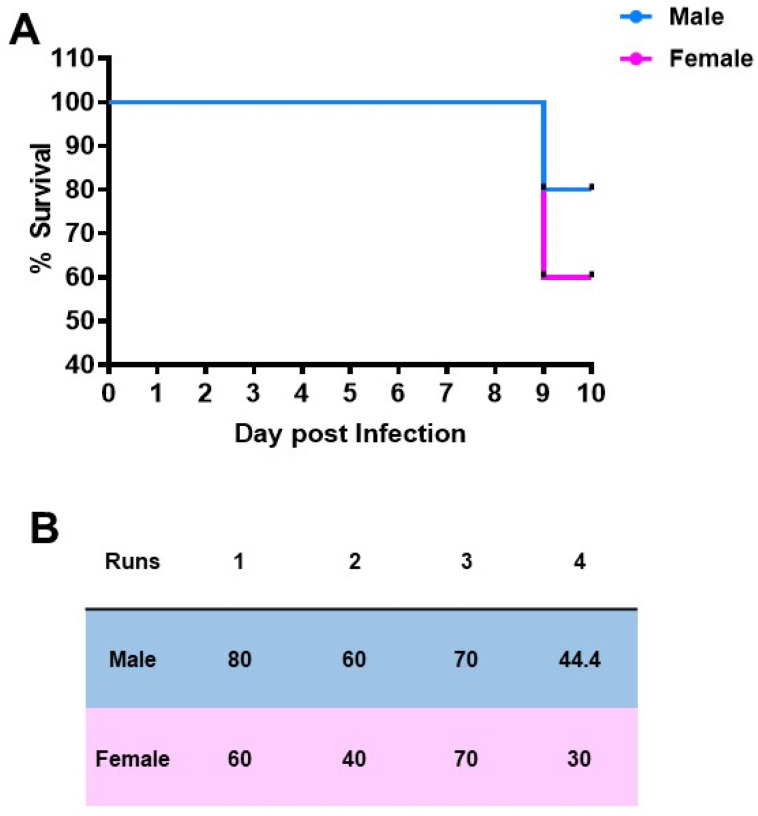
(**A**) Percentage survival of male and female mice infected with *T. gondii* from a representative experiment (**B**). Table showing the percentage survival of four independent experiments analyzed using the ratio paired *t* test (*p* = 0.032). Each value represents the mean of five animals per sex (experiments one and two) or ten animals per sex (experiment three) or nine male and ten female animals (experiment four).

In a total of four separate experiments performed to determine the effects of sex on *T. gondii* infection, female mice had reduced survival compared with male mice. Data from a single representative experiment are shown (Figure 1A). In the series of four replicate experiments are greater percentage survival was noted in male mice compared with female mice (80 versus 60, 60 versus 40, 70 versus 70 and 44.4 versus 30). Data from the four experiments were analyzed using a ratio paired *t* test (*p =* 0.032) (Figure 1B). In addition, female mice suffered weight loss during infection whilst the male mice did not.

### 2.2. Male Mice Have Increased T. gondii Multiplication Compared to Female Mice

**Figure 2:**
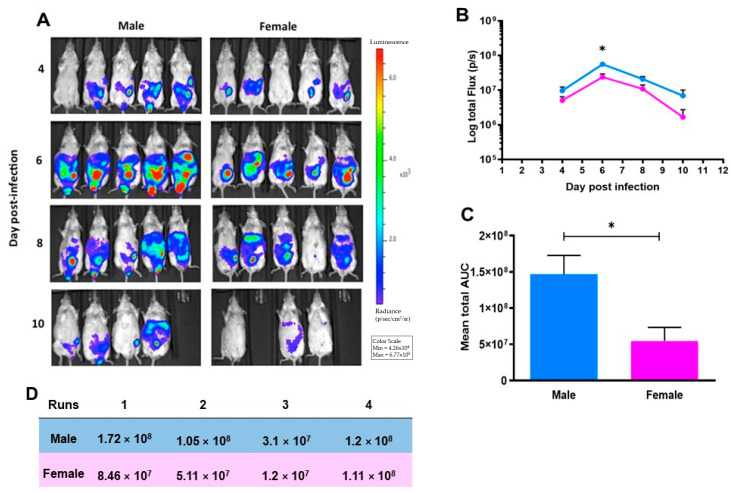
Male mice have increased *T. gondii* multiplication compared with female mice. Data from a representative experiment from 4 separate experiments are shown in A, B, and C. (**A**) Five male and five female mice were infected with 2 × 10^4^ (ip) type II Prugniaud tachyzoites, expressing firefly luciferase. Mice were imaged at 4, 6, 8, and 10-days post-infections. This signal increased during the infection course and its peak was at day six. By day 10, the signal was reduced or no longer detectable in some of mice. (**B**) Quantitative analysis of parasite burdens was significantly higher among males in comparison to females in particularly at six-day post infection when parasite numbers peaked. (**C**) The result of the mean of total area under curve (AUC) of the parasite burden during infection course between male and female groups was significantly higher in male mice than female mice. Each value represents the mean ± SEM of five mice analyzed using a one-tailed nonparametric Mann–Whitney U test * *p* < 0.05. (**D**) The median AUC of four independent experiments using five animals per sex (experiments one and two) or ten animals per sex (experiment three) or nine male and ten female animals (experiment four) (*p* = 0.023).

In a series of 4 separate experiments, 5–10 male and 5–10 female mice (as stated) were infected intraperitoneally with 2 × 10^4^ type II Prugniaud tachyzoites, expressing firefly luciferase and imaged at 4, 6, 8, and 10-bdays post-infections. A bioluminescent signal was visible by day four post-infection, originating from the abdomen of both sexes. Male mice also had a bioluminescent signal in their testicles. Bioluminescence increased in male and female mice over the course of the study, peaking at day six. By day 10 the signal in mice was decreased and, in some cases, was no longer detectable (Figure 2A). Quantitative analysis of parasite burdens was performed by calculating the log total light flux using LivingImage 4.0 software (PerkinElmer, Waltham, MA, USA) and expressed as photons per second. The region of interest (ROI) in the data shown include the testicles. In the representative experiment shown, parasite burden was significantly higher among males in comparison to females, particularly at day six post infection when parasite numbers peaked (Figure 2B). In this experiment, the mean of total Area Under Curve (AUC) of the parasite burden during infection between male and female groups was significantly higher in male mice than female mice (Figure 2C). Similar data was obtained when the region of interest (ROI) was set to remove the signal from the testicles indicating that the observed differences were not due to increased parasite multiplication in this organ. The data from the four individual experiments are detailed in (Figure 2D) and demonstrate that female mice had significantly less parasites (*p* = 0.023) using a ratio paired t test.

### 2.3. Quantitative Analysis of Cytokines Detected in the Plasma of Male and Female Mice Infected with T. gondii

**Figure 3:**
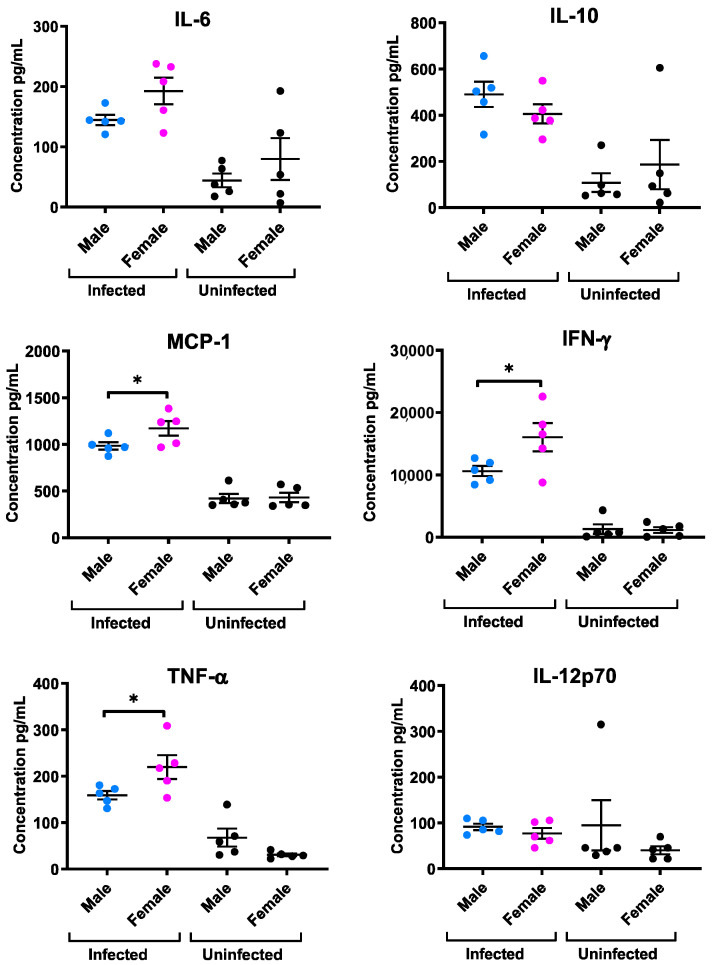
Cytokine concentrations in the plasma of male and female mice infected with *T. gondii.* IL-6, IL-10, MCP-1, IFN-α TNF-α, and IL-12p70 were detected. The levels of these cytokines significantly increased in infected mice compared to uninfected mice. However, in the female group MCP-1, IFN-γ, and TNF-α cytokines were significantly higher in comparison to the male group with (*p* = 0.05, *p* = 0.05, and *p* = 0.03, respectively). Each value represents the mean of five animals per experimental group and were analyzed using one-tailed nonparametric Mann–Whitney analyses ± SEM. * *p* < 0.05.

A cytometric bead array (CBA) was used to simultaneously determine the level of cytokines IL-6, IL-10, MCP-1, IFN-γ, TNF-α, and IL-12p70 in the serum of uninfected mice and mice infected with *T. gondii* at six-days post-infection. Generally, there were significant increases in most cytokine levels within the infected group compared to the uninfected group in both sexes. However, MCP-1, IFN-γ, and TNF-α cytokine levels were greater in infected females compared with infected males (*p* = 0.05, *p* = 0.05 and *p* = 0.03, respectively) (Figure 3). Each value represents the mean of five animals per experimental group and were analysed using one-tailed nonparametric Mann–Whitney analyses ± SEM * *p* < 0.05.

### 2.4. Bone Marrow Macrophages (BMDMs) Characterisation

**Figure 4:**
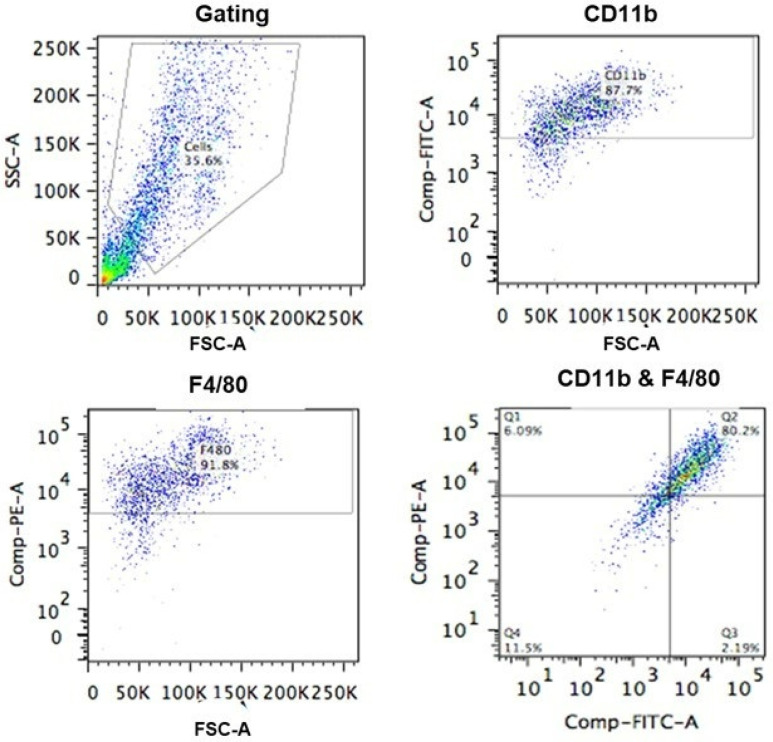
Characterization of BMDMs used in the studies. BMDMs were phenotypically characterized by flow cytometry. Live cells were gated by their forward and side scatter; 91.8% of cells were F4/80 positive and 87.7% of cells were CD11b positive; 80.2% of cells were double positive for F4/80 and CD11b.

BMDMs were obtained from the bone marrow of six to eight-week-old BALB/c male mice. Cells were then cultured in complete DMEM medium supplemented with 20% L929 cells for 10 days to produce BMDMs. The cells then were analyzed to confirm their macrophages phenotype by flow cytometry. For the in vitro experiments, cells at a minimum were 87% CD11b + and 91% of F4/80+. Cells were typically 80% or greater double positive for both markers (Figure 4).

### 2.5. MCP-1 Is Induced by T. gondii Tachyzoites in a Dose Dependent Manner and Is Not Influenced In Vitro by Physiological Levels of Estrogen or Testosterone

**Figure 5:**
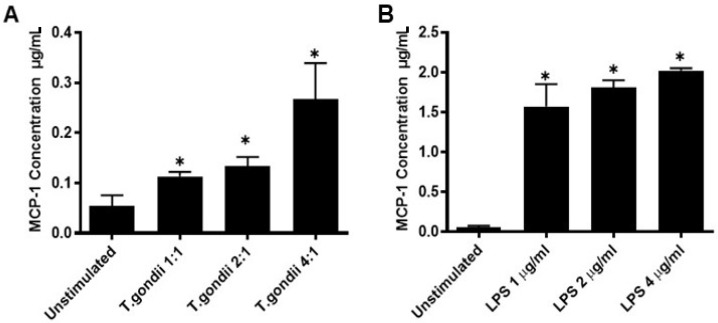
*T. gondii* stimulates macrophage production of MCP-1 in a dose dependent manner (**A**). *T. gondii* induced MCP-1 in a dose dependent fashion. (**B**) LPS induced MCP-1 at concentrations above 2 μg/mL. Each value represents the mean of three replicates and were analyzed using a one-tailed nonparametric Mann–Whitney U test ± SEM * *p* < 0.05.

**Figure 6:**
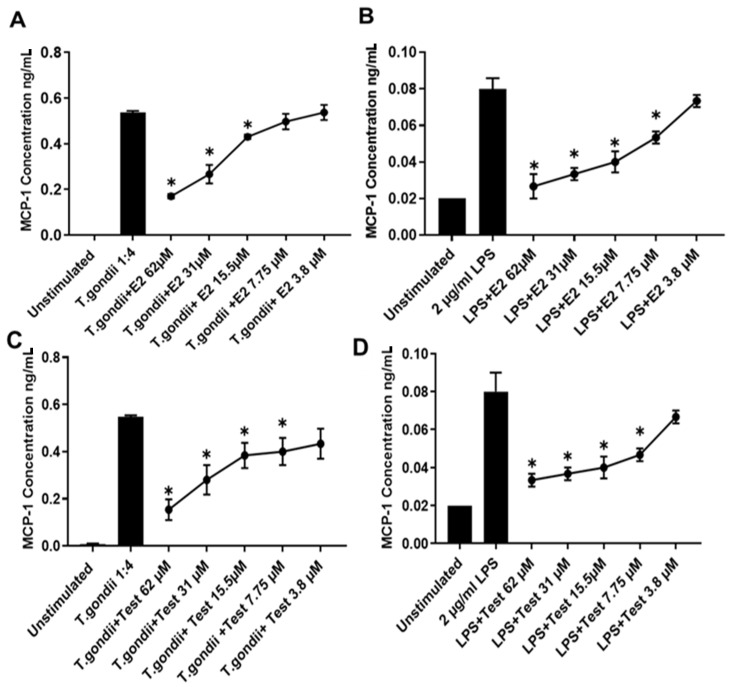
Estradiol 17 beta (*E*2) and Testosterone downregulate MCP-1 production in a dose-dependent manner. Macrophages were treated with a range of concentrations of Estradiol (*E*2) then infected (**A**) *T. gondii* or stimulated with (**B**) LPS. Macrophages were also treated with a range of concentrations of Testosterone then infected with (**C**) *T. gondii* or stimulated with (**D**) LPS. Both hormones significantly reduced MCP-1 production in a dose dependent manner. Each value represents the mean of three replicates and were analyzed using a one-tailed nonparametric Mann–Whitney U test ± SEM * *p* < 0.05.

Macrophages were incubated with different numbers of T. gondii tachyzoites to determine the potential relationship between MCP-1 production and parasite load. MCP-1 was found to be induced in a dose dependent manner with higher numbers of tachyzoites inducing higher levels of cytokine (Figure 5A). A dose reponse was also obtained with LPS (Figure 5B). This confirmed that the increased MCP-1 levels noted in the serum of female mice, relative to male mice was due to an intrinsic sex difference in MCP-1 production. The ability of estrogen and testosterone to modulate levels of MCP-1 in macrophages cultured with tachyzoites were examined. Both hormones significantly reduced MCP-1 production in a dose dependnet manner, but only at relatively high pharmacological levels (Figure 6C,D).

## 3. Discussion

Studies of a wide variety of inbred mice of diverse genetic backgrounds and major histocompatibility complex haplotypes found a difference in the susceptibilities of males and females to *T. gondii* infection [12]. Roberts et al. (1995) demonstrated that female mice from a wide variety of strains exhibited greater levels of mortality and weight loss than their male counterparts [12]. However, enumeration of parasite burdens during the acute phase of infection was not easy to achieve and thus not reported in these early studies. To overcome this, this study used *T. gondii* genetically engineered to express the luciferase gene to allow facile quantification using an in vivo imaging system (IVIS). Female BALB/c mice infected with these genetically altered parasites were confirmed to be more susceptible to acute infection, as determined by higher mortality rates and weight loss. However, importantly and contrary to expectations, the current studies demonstrate that male mice had higher parasite burdens during the acute infection despite their lower mortality. The reasons for this and the contribution of the immune response was therefore examined in more depth.

Plasma collected from experimental animals at day six post-infection was used to determine cytokine production during *T. gondii* infection. Results showed that infected male and female mice exhibited a significant increase in levels of most of the examined cytokines, namely IL-6, IL-10, MCP-1, IFN-γ, TNF-α, and IL-12p70, compared to the uninfected group. However, pertinent to the sex differences found in *T. gondii* immunity, MCP-1, IFN-γ, and TNF-α concentrations were found to be significantly higher in plasma from infected female mice than male mice. It is well known that these cytokines play a key role in the pathogenesis of toxoplasmosis. The induction of a type 1 inflammatory response with IFN-γ and TNF-α is known to be important during the early stages of immunity to *T. gondii* [16]. IFN-γ and TNF-α production stimulates macrophage functions and controls tachyzoite replication during acute and chronic phases of infection [17]. IFN-γ is the main mediator of resistance to *T. gondii* and stimulates multiple intracellular mechanisms to kill the parasite and inhibit its replication [18]. Many studies of mice deficient in IFN-γ demonstrate that they are susceptible to *T. gondii* infection and fail to control parasite burden [19]. Consistent with the higher levels of IFN-γ in female mice infected with *T. gondii*, female C57BL/6 mice when inoculated with *Plasmodium chabaudi* exhibited higher expression of IFN-γ compared to infected male mice [20].

TNF-α also plays a significant role in modulating the immune response during *T. gondii* infection [21]. TNF-α is critical for controlling disease and often acts in concert with IFN-γ to restrict parasite replication. Furthermore, TNF-α and IFN-γ, through stimulation of nitric oxide have been shown to contribute to induction of stage conversion and the formation of tissue cysts [22]. TNF-α is also an important factor for control of *T. gondii* in vivo and survival of acute and chronic murine toxoplasmosis as demonstrated by the inability of TNF knockout mice to control intracerebral *T. gondii* and succumbing to an acute necrotizing toxoplasma encephalitis. Furthermore, mice deficient in the TNF receptor subunit (TNFRp55) are more susceptible to intraperitoneal infection with *T. gondii* and develop increase parasite numbers in their liver, lung and spleen [23]. In keeping with the increased levels of TNF-α in female mice infected with *T. gondii*, a recent study found that when compared with men, women respond to bacterial endotoxin with greater pro-inflammatory responses including significantly higher levels of TNF-α [24].

Monocyte chemoattractant protein 1 MCP-1/CCL2 is known to be produced during the early phase of *T. gondii* infection. Mice deficient in the CCR2 chemokine receptor (its ligand), fail to recruit monocytes and succumb to infection. Both CCR2-/- and MCP-1-/- mice show amplified mortality and pathology in response to oral *T. gondii* challenge [25]. Thus, the increased levels of MCP-1 found in the serum of female mice relative to male mice infected with *T. gondii* could contribute to the reduced parasite numbers seen in female mice. In vitro studies found that physiological levels of estrogen did not increase MCP-1 production by macrophages stimulated with LPS or co-cultured with tachyzoites. Furthermore, pharmacological levels of estrogen actually reduced MCP-1 production and testosterone only reduced MCP-1 levels in these cultures when used at pharmacological concentrations. These results suggest that MCP-1 is not directly affected by estrogen or testosterone at normal physiological levels and that more complex sex-dependent factors must be responsible for the observed sex differences in vivo. Further studies will be necessary to show the role of both hormones in the regulation of cytokine production in mice infected with *T. gondii.* Notably, testosterone has been demonstrated to have systemic anti-inflammatory effects in a number of human disease states [26]. Characterization of hormone receptor expression in bone-marrow-derived macrophages could also be helpful to understand potential hormone-mediated mechanisms responsible for modulation of cytokine production.

Sex differences in MCP-1/CCL2 have been found in macaques, with females having significantly higher levels of MCP-1/CCL2 in their plasma compared to their male counterparts during HIV infection. The elevated levels of MCP-1 in female macaques were surprising given the lower viral loads in HIV infected women reported in earlier studies [27]. One possibility is that difference observed between males and females in serum MCP-1 production following infection is due to sex-dependent differences in monocyte trafficking.

In conclusion, the studies described herein suggest that female mice respond to *T. gondii* infection with greater inflammatory cytokine production than male mice. The increased level of these cytokines in female mice may explain why they had less parasite burden during the acute stage of *T. gondii* infection than male mice as measured by IVIS. However, the raised levels of these inflammatory cytokines might also contribute to disease manifestations and explain why they also suffered more in terms of higher mortality rates and severe disease outcomes. There is now abundant evidence about the major role cytokines can play in the pathogenesis of *T. gondii,* and it is also well known that these cytokines can play protective and detrimental roles during infection. Therefore, a balance in their production is critical to maintain their benefits in controlling infection while minimizing the pathological impact on the host [28]. Similar observations have been made in mice infected with influenza viruses, wherein females have reduced viral load and increased inflammation [29]. Understanding how sex impacts on the prognosis of infectious diseases is important for optimizing treatments. Specifically, it is important to know if poor prognosis is due to an inability to control the infection or the accompanying immune response. The challenge in treating the immune response will be to do so in a manner that does not benefit pathogen replication.

## 4. Materials and Methods

### 4.1. Mice

Experimental groups contained 5–10 mice per group. BALB/c mice were bred in house at the Strathclyde Institute of Pharmacy and Biomedical Sciences, Glasgow, UK. Mice were maintained under a 12-h light-12 hours’ dark period. For experimental groups, male and female mice were used between 8-11 weeks old. During the experimental course the animals were weighed daily and scored for signs of illness. Animals exceeding humane thresholds were euthanized.

### 4.2. T. gondii Maintenance

The *Prugniaud T. gondii* type II strain genetically modified to express the Firefly Luciferase (FLUC) gene was utilized [30]. Tachyzoites were routinely maintained in confluent human foreskin fibroblasts (HFFs) grown in DMEM complete medium comprising of 10% fetal calf serum, 5 mM L-glutamine, 100 U/mL penicillin, 100 μg/ mL streptomycin and 50 U/mL amphotericin B at 37 °C in 5% CO_2_.

### 4.3. Infection Mice with T. gondii

Each mouse was intraperitoneally injected with 2 × 10^4^ tachyzoites of *T. gondii* in 400 μL of PBSs.

### 4.4. In Vivo Imaging and Set Up IVIS Spectrum 200 Series

Starting on the fourth day post infection in vivo imaging was carried out using the IVIS Spectrum 200 Series. The group being imaged was injected intraperitoneally with 200 μL of 15 mg/mL d-luciferin potassium salt (PerkinElmer Lifesciences) solution prior to imaging experiments. Mice were anaesthetized with a 2.5%–3.5% isoflurane/oxygen mix. Once the animals were anaesthetized, they were relocated to the imaging chambers ventrally to the nose cones that attached to the manifold inside the imaging chamber so that anesthesia could be maintained. At 20 minutes’ post-injection with d-Luciferin imaging was carried out with a 1-min exposure. This was shown to establish the pe91ak of the bioluminescent signal following conducting of d-luciferin solution [31].

### 4.5. Cytokines Measurement in Plasma Collected during Acute Infection

To measure the cytokines level in blood samples, cardiac puncture procedure was applied to collect blood samples. All sacrificed procedures were done under Schedule 1 of Animal Scientific Procedures Act 1986. Interferon gamma (IFN-γ), Monocyte chemoattractant protein 1 (MCP1) Interleukin 12 (IL-12p70), Interleukin 10 (IL-10), Interleukin 6 (IL-6), and tumor necrosis factor alpha (TNF-α) by using the BD Cytometric Bead (Biosciences.BD, UK). The blood samples were collected from infected male and female mice at day 6 post-infection in addition to uninfected male and female mice. Following the manufacturer’s recommended protocol, the assay was carried out. Acquisition was undertaken using a BD FACSDiva™ software. (BD Biosciences, Oxford, UK), and results determined using Kaluza 1.3 Analysis Software (Beckman Coulter, High Wycombe, UK).

### 4.6. Producing Mouse Bone Marrow-Derived Macrophages (BMDMs) 

Bone marrow cells were cultured from the femur and tibia bones of six to eight-week-old BALB/c male mice. The mouse was sacrificed by cervical dislocation. The femur and tibia removed and cleaned from adherent tissues and washed with 70% ethanol. The bones were cut at both ends and the bone marrow obtained inserting a 15-gauge needle into the cavity and flushing each bone with complete Dulbeccos Modified Eagle Medium (DMEM) (Gibco, Thermo Fisher Scientific, Paisley, UK) containing of 20% Fetal Calf Serum (FCS) (Gibco, Thermo Fisher Scientific, Paisley, UK), 2% Penicillin/streptomycin solution (Sigma-Aldrich, Poole, UK), 2% l-Glutamine (Gibco, Thermo Fisher Scientific, Paisley, UK). The eluted cells then collected, filtered using a 40 μm cell strainer (Thermo Fisher Scientific, Paisley, UK) and centrifuged at 420× *g* for 5 min. After that the supernatant was discarded the pellet resuspended in 10mL per bone of complete DMEM medium with addition of 30% of L cell conditioned media (containing Macrophage-colony stimulating factor (M-CSF) was added to the cell pellet. The cells were cultured in 10 cm^2^ Petri dishes, 10 mL of cells per plate. The dishes were maintained at 37 °C in humidified conditions and 5% (*v*/*v*) CO_2_. On day three 10 mL of fresh complete DMEM was added to feed the macrophages. On day seven all of the media was replaced with 20 mL fresh complete DMEM containing 30% L cell conditioned media. On day ten the cells were harvested by adding 5 mL ice cold medium (Gibco, Thermo Fisher Scientific, Paisley, UK) and scraping them to allow adherent cells to detached. The cell suspension was centrifuged at 420× *g* for 5 min. The pellet was resuspended in complete RPMI and cell count and was determined using trypan blue (Sigma, Poole, Dorset, UK). The cell concentration was adjusted to 2 × 10⁶ cells/mL. The phenotype of the cells was characterised using flow cytometry. Cells were plated in 96 well plates (TPP, Trasadingen, Switzerland) at 2 × 10^5^ cells/well.

### 4.7. Flow Cytometry

0.5 × 10^6^ cells were resuspended in 100 μL FACS Buffer (2% Bovine Serum Albumin (Sigma, Dorset, UK) in PBS (Gibco, Thermo Fisher Scientific, Paisley, UK) containing specific anti-CD11b (FITC) (BD Pharmingen, San Diego, CA, USA) and anti F4/80 (PE) (eBioscience) antibodies at 1/100 dilution and incubated for 30 min on ice, in the dark. Single stain and unstained controls were set up in parallel. Following incubation samples were washed with 5ml FACS Buffer and centrifuged at 300× *g* for 6 min. This was repeated a further two times. After the final wash the cells were resuspended in 300 μL FACS Buffer for analysis. Analysis was carried out using BD FACS Canto II running FACSDiva immunocytometry system (BD Pharmingen). Voltages and system compensation were set up using unstained and single stain controls prior to samples being ran. Samples were run at a medium flow rate and 20,000 events collected per sample. Analysis was carried out using FlowJo software (Version 10.7, BD biosciences, New York, NY, USA) to calculate cell populations positive for CD11b and F4/80. Cells were typically 80% or greater double positive for CD11b and F4/80.

### 4.8. Statistical Analyses

Statistical analyses were performed by Prims 8 statistical analysis software (Graphpad Prism, San Diego, CA, USA).

## Data Availability

The data that support the findings are openly available through the University of Strathclyde pureportal at: https://doi.org/10.15129/108ef43f-accc-4de5-8a36-edadc5987810.
